# Three-dimensional assessment of facial asymmetry in class III subjects, part 2: evaluating asymmetry index and asymmetry scores

**DOI:** 10.1007/s00784-023-05193-x

**Published:** 2023-08-24

**Authors:** Deepal Haresh Ajmera, Congyi Zhang, Janson Hoi Hei Ng, Richard Tai‑Chiu Hsung, Walter Yu Hang Lam, Wenping Wang, Yiu Yan Leung, Balvinder S. Khambay, Min Gu

**Affiliations:** 1 Discipline of Orthodontics, Division of Paediatric Dentistry and Orthodontics, Faculty of Dentistry, the University of Hong Kong, Hong Kong SAR, China; 2 Department of Computer Science, The University of Hong Kong, Hong Kong SAR, China; 3 Faculty of Dentistry, the University of Hong Kong, Hong Kong SAR, China; 4 Department of Computer Science, Hong Kong Chu Hai College, Hong Kong SAR, China; 5 Division of Oral and Maxillofacial Surgery, Faculty, of Dentistry, the University of Hong Kong, Hong Kong SAR, China; 6 Discipline of Prosthodontics, Faculty of Dentistry, the University of Hong Kong, Hong Kong SAR, China; 7 Texas A&M University, College Station, TX USA; 8 Institute of Clinical Sciences, College of Medical and Dental Sciences, The School of Dentistry, University of Birmingham, Birmingham, UK

**Keywords:** Facial asymmetry, 3D, Three dimensional, Asymmetry index, Asymmetry scores

## Abstract

**Objectives:**

To evaluate the outcomes of corrective surgical treatment for craniofacial asymmetry using four different methods with the aim of developing the best technique for craniofacial asymmetry assessment.

**Materials and methods:**

CBCT images of twenty-one class III subjects with surgically corrected craniofacial asymmetry and twenty-one matched controls were analyzed. Twenty-seven hard tissue landmarks were used to quantify asymmetry ﻿using the following methodologies: the asymmetry index (AI), asymmetry scores based on the clinically derived midline (CM), Procrustes analysis (PA), and modified Procrustes analysis (MPA).

**Results:**

Modified Procrustes analysis successfully identified pre-operative asymmetry and revealed severe asymmetry at the mandibular regions compared to controls, which was comparable to the asymmetry index and clinically derived midline methods, while Procrustes analysis masked the asymmetric characteristics. Likewise, when comparing the post-surgical outcomes, modified Procrustes analysis not only efficiently determined the changes evidencing decrease in facial asymmetry but also revealed significant residual asymmetry in the mandible, which was congruent with the asymmetry index and clinically derived midline methods but contradictory to the results shown by Procrustes analysis.

**Conclusions:**

In terms of quantifying cranio-facial asymmetry, modified Procrustes analysis has evidenced to produce promising results that were comparable to the asymmetry index and the clinically derived midline, making it a more viable option for craniofacial asymmetry assessment.

**Clinical relevance:**

Modified Procrustes analysis is proficient in evaluating cranio-facial asymmetry with more valid clinical representation and has potential applications in assessing asymmetry in a wide spectrum of patients, including syndromic patients.

## Introduction

Aesthetic faces have been proven to influence individuals’ personality development, which can dictate their social, economic, and health status [1, 2]. In tandem with sexual dimorphism, juvenescence, and uniformity, symmetry is also a prerequisite for an attractive face [2, 3]. However, perfect symmetry in humans continues to be a hypothetical concept [4, 5], thus compelling individuals toward reconstructive surgical procedures to restore facial harmony [2, 6]. Nevertheless, the effect of surgical treatment is directly reliant on the precision of the diagnosis, thereby making accurate evaluation an essential and mandatory step before planning the surgical procedure.

The advent of 3-dimensional (3D) imaging modalities has provided additional diagnostic tools for clinical use [7]. Accurate and comprehensive knowledge of facial discrepancies can be obtained from 3D images, as it is possible to rotate and view 3D images from different angles [8]. There are numerous methods documented in the literature to quantify facial asymmetry. Several studies have calculated asymmetry 3-dimensionally by measuring linear, angular, and surface distances of several landmarks from the plane of symmetry [9–12]. In addition, others have performed surface area and volumetric measurements [6]. Furthermore, some studies have also used a 3D image-based coordinate assessment approach to compare assorted symmetry factors (region-based asymmetry index) [4, 13, 14], while other studies have calculated asymmetry scores [2, 15].

A midsagittal plane (symmetry plane) is central for the quantification of facial asymmetry and can be constructed by manually selecting the reference landmarks not affected by the asymmetry [6, 16–18] or by deriving it mathematically based on a best-fit superimposition method called ﻿“*Procrustes analysis*.” Several studies have analyzed facial asymmetry using clinical midline and Procrustes methods; however, flaws associated with these methods, such as unreliability and falsified presentation of true asymmetric features, have also been reported [2, 19], which might influence the diagnosis and post-operative treatment outcomes. Hence, a method that yields reliable evaluation of asymmetry is required. Therefore, the present study aimed to compare four different methods and develop the best technique for the assessment of facial asymmetry.

## Materials and method

### Sample size calculation

By considering the mean difference of 0.66 mm (standard deviation of 0.5) as clinically significant [14], together with a power of 95%, an effect size of 1.32, and alpha level set at 0.05, a minimum sample size of 32 (16 in each group) was calculated with G*Power (version 3.1.9.2, Kiel University, Germany) [20].

### Subjects

#### Asymmetry group

Twenty-one subjects (7 male and 14 females) aged 18 to 40 years (23.0 ± 3.4 years) from the orthodontic-orthognathic pool who sought surgical treatment at the Prince Philip Dental Hospital, University of Hong Kong, between April 2012 and July 2019 were chosen if they fulfilled the following inclusion criteria: (1) soft tissue chin deviation > 3 mm, (2) bimaxillary surgery with no genioplasty, (3) pre-operative cone-beam computed tomography (CBCT) scan (*T*_0_) and post-surgical CBCT scan (*T*_1_) taken at least 6 months after surgery, (4) had no history of craniofacial syndromes or craniofacial surgery, and (5) were not diagnosed with hemifacial microsomia or orbital dystopia.

#### Control group

Twenty-one age (23.0 ± 3.3 years) and gender (7 males and 14 females) matched subjects from the same hospital who had taken CBCT scans in 2015 for nonsurgical purposes and who satisfied the following inclusion criteria were recruited for the present study. Inclusion criteria: (1) imperceptible maxillo-mandibular asymmetry (soft tissue chin deviation < 3 mm), (2) class I skeletal pattern, (3) decently aligned dental arches, and (4) no previous record of temporomandibular disorder, craniofacial syndromes, or craniofacial surgery. Table 1 represents the baseline characteristics of the subjects in the asymmetry and control groups.

**Table 1:** Patient characteristics in the asymmetry and control groups

Group	Sex			Age (years)	Surgery			Me deviation (mm)
	Male (*n*)	Female (*n*)	Total (*n*)	Mean ± SD	Le Fort I + BVSO^a^	Le Fort I + BSSO^b^	Le Fort I + VSO^c^ + SSO^d^	Mean ± SD
Asymmetry group	7	14	21	23.0 ± 3.4	13	6	2	7.31 ± 4.10
Control group	7	14	21	23.0 ± 3.3	–	–	–	1.22 ± 0.80

^a^ BVSO, bilateral vertical subsigmoid osteotomy

^b^ BSSO, bilateral sagittal split osteotomy

^c^ VSO, vertical subsigmoid osteotomy

^d^ SSO, sagittal split osteotomy

#### CBCT acquisition

Each patient was scanned using *ProMax 3D Mid* (Planmeca, Helsinki, Finland) with the following parameters: 90 kVp, 400 μm voxel size, 4.7 s scan time, and 20 cm × 17 cm field of view. Each subject was seated with the head positioned such that the Frankfurt horizontal (FH) plane was parallel to the ground while maintaining a mild contact of the lips to their teeth and labio-mental soft tissue at rest throughout the scanning process. CBCT scans were saved in Digital Imaging and Communications in Medicine (DICOM) format and then imported to 3D Slicer 4.10, an open-source medical image processing software platform (http://www.slicer.org) for analysis [21].

### Analysis of asymmetry

Four different methodologies, the asymmetry index (AI) [4, 13, 14, 22] using the landmark-based midsagittal plane, the asymmetry scores using the clinically derived midline (CM) [2], Procrustes analysis (PA) [2, 23], and our new-found technique, modified Procrustes analysis (MPA), were utilized to evaluate the results of corrective surgical treatment of facial asymmetry in class III patients compared with controls.

### 3D model generation, registration, and 3D analysis

**Fig. 1:**
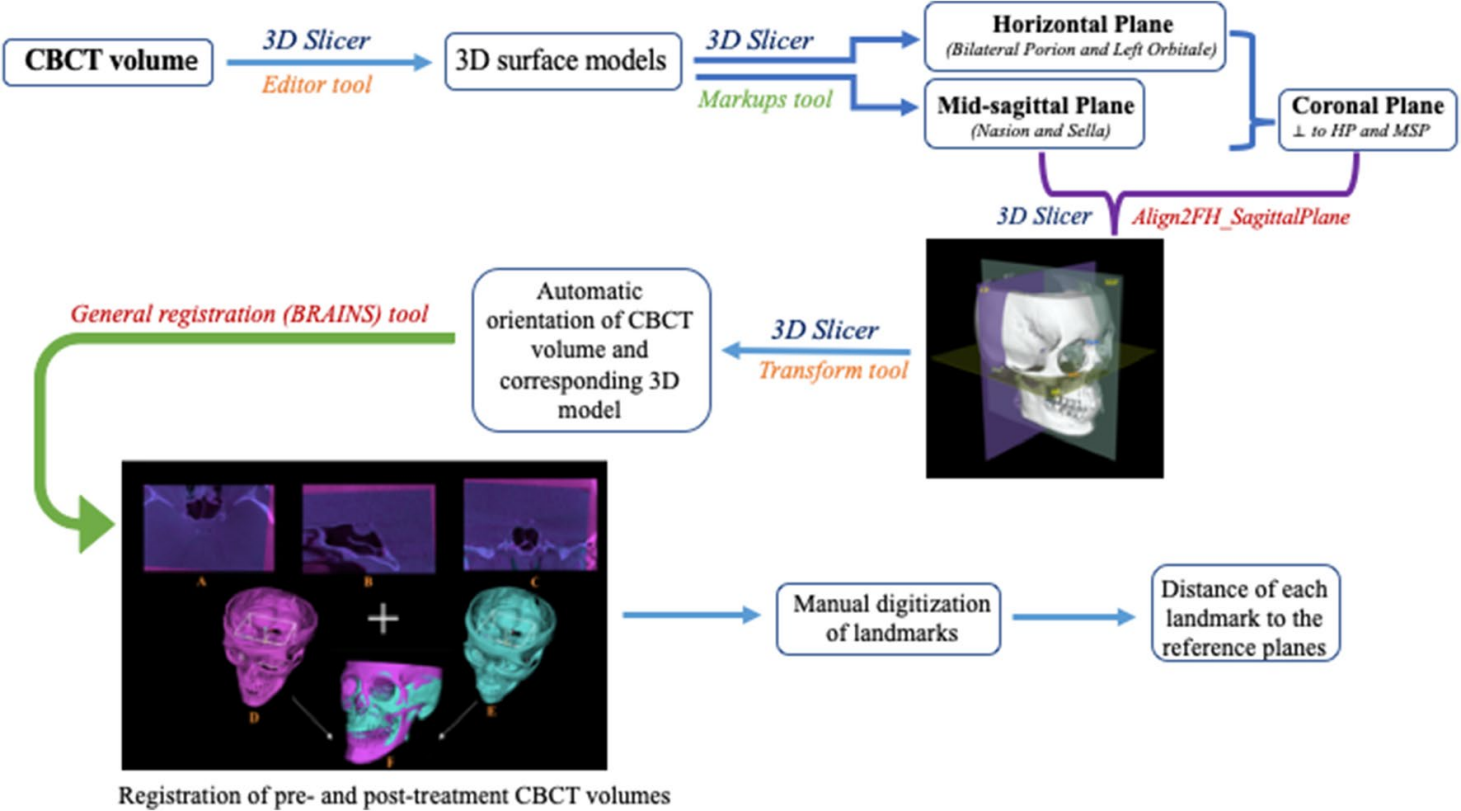
Steps in 3D model generation, registration, and 3D analysis

A detailed description of the landmarks and reference planes [6, 23–27] utilized in the current research is presented in Table 2. 3D surface models were generated from the CBCT volumes for each patient through bone segmentation using 3D Slicer’s “*Editor tool*.” Next, “*Markups tool*” enabled manual digitization of bilateral orbitale and left porion landmarks, to establish the horizontal plane (HP), while the nasion and sella to define the midsagittal plane (MSP) perpendicular to the HP. Coronal plane (CP) was built passing through the left porion and perpendicular to the HP and MSP. Using slicer extension “*Align2FH_SagittalPlane*,” the HP plane was aligned along the *x*–*z* plane, and MSP was aligned along the *y*–*z* plane, while the porion was set to lie on the *x*-axis. Thereafter, the CBCT volume and its corresponding 3D model underwent automatic reorientation via the “*Transform tool*” appertaining to the reference planes mentioned above. Later, using a 2-step semiautomated registration technique, pre-operative (*T*_0_) and post-operative (*T*_1_) CBCTs of each patient were superimposed based on selecting the region of interest (ROI) involving predetermined stable cranial structures unaffected by the surgery. Detailed methodology for orientation and registration of CBCT volumes can be referred from the previously published study [28]. Subsequently, 20 bilateral and 7 midline landmarks (Table 2) were identified on CBCT scans and digitized manually on the 3D reconstructed models of *T*_0_, *T*_1_ and control patients and the distance of each landmark to the three reference planes was measured as *d*_R_, *d*_A_, and *d*_S_ in millimeters (mm) (Fig. 1).

**Table 2:** Definitions of landmarks and reference planes used in the study

S. no		Landmarks	Abbreviation	Definition	Reference Author, year
1	Midline landmarks	Anterior nasal spine	ANS	Tip of the anterior nasal spine of the palatal bone in the hard palate	Jung et al. 2009[20]
2		Pt A	Pt A	The point of maximum concavity on the contour of premaxilla below ANS	Damstra et al. 2012[19]
3		Upper incisor midpoint	UIM	Contact point between the upper central incisors	Jung et al. 2009[20]
4		Lower incisor midpoint	LIM	Contact point between the lower central incisors	Jung et al. 2009[20]
5		Pt B	Pt B	The point of maximum concavity on the midline on the alveolar process of mandible	Leung et al. 2018[22]
6		Pogonion	Pog	The most anterior point in the symphysis	Jung et al. 2009[20]
7		Menton	Me	The most inferior point in the symphysis	Jung et al. 2009[20]
8	Bilateral landmarks	Infra orbital foramen	IOF	The external opening of the infra orbital canal, on the anterior surface of the body of maxilla in the right and left sides	
9		Zygion	Zyg	Most anterior, lateral point on the zygomatic arch in frontal view in the right and left sides	Ercan et al. 2013[21]
10		Canine fossa	CF	A depression on the anterior surface of the maxilla below the infraorbital foramen and on the lateral side of the *canine* eminence in the right and left sides	
11		Pyriform aperture	PA	The most concave point on pyriform aperture	
12		Lowest pyriform Aperture	LPA	The lower most point on the concavity of the pyriform aperture	
13		Maxillary tuberosity	MT	Point of maximum convexity on the maxillary alveolar ridge in the right and left sides	
14		Convex point on zygoma	Cx Z	The most convex part of the zygomatic bone (malar) in the lateral view	
15		upper canine	UC	The most prominent point on the buccal surface of the upper canine	Leung et al. 2018[22]
16		Lower canine	LC	The most prominent point on the buccal surface of the lower canine	Leung et al. 2018[22]
17		Upper 1^st^ molar	UM1	Mesio-buccal cusp of upper 1^st^ molar in the right and left sides	Leung et al. 2018[22]
18		Lower 1^st^ molar	LM1	Mesio-buccal cusp of lower 1^st^ molar in the right and left sides	Leung et al. 2018[22]
19		Mental foramen	MF	Anterior opening of the mandibular canal on the body of the mandible lateral to and above the *mental* tubercle in the right and left sides	Suzuki-Okamura et al. 2015[23]
20		Lateral chin points	CP	The most anterior point of chin on the outline of mandibular symphysis at lower canine region in the right and left sides	Leung et al. 2018[22]
21		Gonion lateralis	GoL	Most lateral point between the mandibular corpus and the ramus junction in the right and left sides	Nur et al. 2016 [6]
22		Gonion inferius	GoI	Most inferior point between the mandibular corpus and the ramus junction in the right and left sides	Nur et al. 2016[6]
23		Gonion posterius	GoP	Most posterior point between the mandibular corpus and the ramus junction in the right and left sides	Nur et al. 2016[6]
24		Antegonial notch	AGo	Deepest point of the concavity between the mandibular corpus and the ramus junction in the right and left sides	Nur et al. 2016[6]
25		Condylar	Con	Most superior midpoint of the condylar head in the right and left sides	Nur et al. 2016[6]
26		coronoid	Crn	The most superior point of right coronoid process in the right and left sides	Leung et al. 2018[22]
27		Sigmoid notch	Sig	The depth of concavity at right sigmoid notch in the right and left sides	Leung et al. 2018[22]
28		Orbitale	Or	The most inferior point of the lower margin of the bony orbit in the right and left sides	Damstra et al. 2012[19]
29		Porion	Por	The most superior point of the external auditory meatus in the right and left sides	Leung et al. 2018[22]
30		Nasion	Na	Midpoint of the frontonasal suture	Nur et al. 2016[6]
31		sella	S	Center of the hypophyseal fossa	Nur et al. 2016 [6]
		Reference Planes			
1		Horizontal plane	HP	A plane passing through the bilateral orbitales and right porion	Nur et al. 2016[6]
2		Mid-sagittal plane	MSP	A plane perpendicular to HP and passing through the nasion and sella	Nur et al. 2016[6]
3		Coronal plane	CP	A plane perpendicular to the HP and MSP and passing through the right porion	Nur et al. 2016[6]

### Asymmetry index (AI)-based analysis

To assess facial asymmetry, a region-based AI was created by summing each landmark AI for that region (Table 3). AI must approach zero for a perfectly symmetrical face. The AI for various regions was calculated as follows [14]:

$$\begin{array}{l}\mathrm{AI}\;\mathrm{of}\;\mathrm{facial}\;\mathrm{midline}\;\mathrm{landmarks}={\mathrm d}_{\mathrm R};\\\mathrm{AI}\;\mathrm{of}\;\mathrm{bilateral}\;\mathrm{landmarks}=\surd{({\mathrm{Dd}}_{\mathrm R}-{\mathrm{Nd}}_{\mathrm R})}^2+{({\mathrm{Dd}}_{\mathrm A}-{\mathrm{Nd}}_{\mathrm A})}^2+{({\mathrm{Dd}}_{\mathrm S}-{\mathrm{Nd}}_{\mathrm S})}^2,\end{array}$$

where *D* = deviated side and *N* = non-deviated side.

**Table 3:** Regionwise summations of the landmarks for the assessment of asymmetry

Zone asymmetry	Landmarks*
Total facial skeleton	IOF, Zyg, CF, PA, LPA, MT, CxZ, MF, CP, GoP, GoI, GoL, Ago, Crn, Sig, Con, ANS, Pt A, Pt B, Pog, Me
Total maxilla	IOF, Zyg, CF, PA, LPA, MT, CxZ, ANS, Pt A
Total mandible	MF, CP, GoP, GoI, GoL, Ago, Crn, Sig, Con, Pt B, Pog, Me
Part asymmetry	
Maxilla midline	ANS, Pt A
Maxilla bilateral	IOF, Zyg, CF, PA, LPA, MT, CxZ
Mandibular midline	Pt B, Pog, Me
Mandibular bilateral	MF, CP, GoP, GoI, GoL, Ago, Crn, Sig, Con
Area asymmetry	
Chin	Pog, Me, CP
Ramus	GoP, GoI, GoL, Ago, Crn, Sig, Con

^*^Refer to Table 2 for definition of the landmarks

### Clinically derived midline based analysis (CM)

**Fig. 2:**
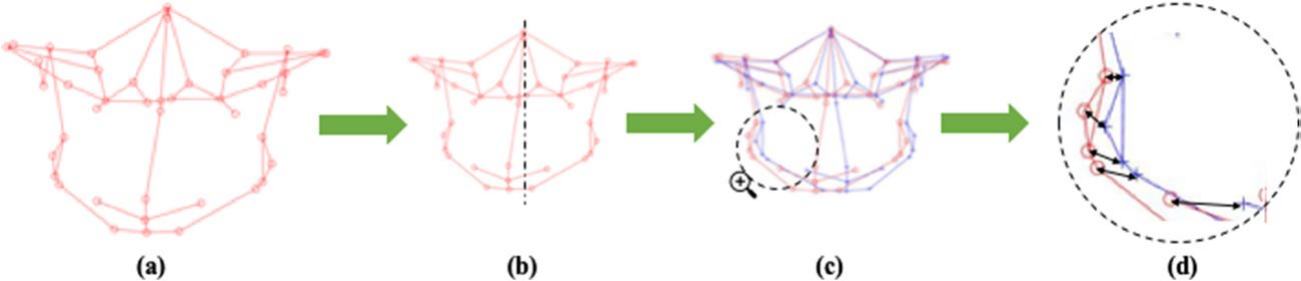
Steps in clinically derived midline-based analysis (CM): **a** original 3D landmark configuration with; **b** original 3D configuration rescaled to a common centroid size and mirrored around the clinically derived midline (dashed line) derived from Nasion, Sella, and perpendicular to the horizontal plane; **c** original configuration (in red) and reflected configuration (in blue); **d** black arrows depicting Euclidean distance between each pair of landmarks (original configuration in red and its reflected configuration in blue)

For each individual, the comma-separated value files (.CSV file) of the 3D landmark coordinates including the coordinates (Nasion and Sella) for generating the “clinically derived midline” were imported to “﻿*MATLAB*” (The MathWorks, Inc., USA). Next, the 3D 27-landmark configuration (﻿original configuration) along with a “clinically derived midline” (perpendicular to horizontal plane) was generated. Following this, the centroid (geometric center) for each 3D configuration was determined, and scaled to a common centroid size, which was then used to generate ﻿a “reflected” 3D landmark configuration by mirroring the 3D ﻿original configuration about the “clinically derived midline.” Thereafter, “Euclidean” distances between each pair of landmarks (original landmark and its reflected configuration) were measured (Fig. 2). Facial asymmetry evaluation was performed by dividing the face into 9 regions (Table 3), and region-wise asymmetry scores were computed for each region by squaring the Euclidean distances between each pair of landmarks and then summing and dividing it by the total number of landmarks assigned to that region. This procedure was repeated for each subject in the asymmetry and control groups. The greater the discrepancy between the landmarks and their reflected configuration, the higher the asymmetry scores, which signifies the severity of facial asymmetry.

### Procrustes analysis (PA)

**Fig. 3:**
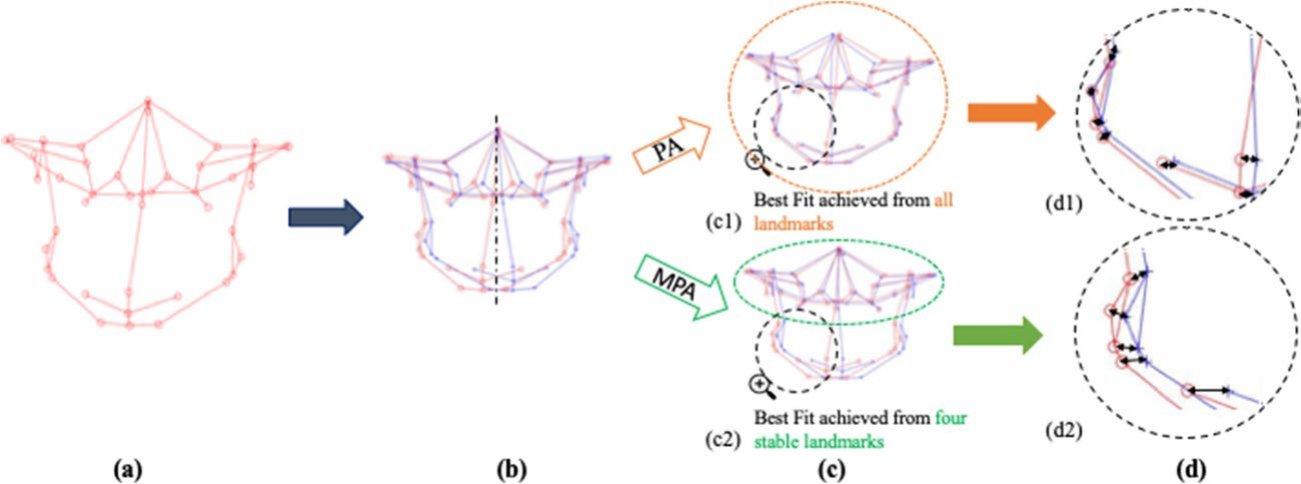
Steps in Procrustes analysis (PA) and modified Procrustes analysis (MPA): **a** original 3D landmark configuration; **b** original 3D configuration (in red) rescaled to a common centroid size and then mirrored about arbitrary midline plane (dashed line); reflected configuration in blue; **c** superimposition of the original configuration (red) with the reflected configuration (blue); **c1** PA utilizes all the landmarks (depicted with orange circle) to achieve best fit; **c2** MPA utilizes only four stable landmarks (depicted with green circle) to achieve best fit; **d** black arrows depicting Euclidean distance between each pair of landmarks (original configuration in red and its reflected configuration in blue); **d1** Euclidean distances in PA; **d2** Euclidean distances in MPA

The 3D landmark configuration (original configuration), as mentioned previously, was imported into ﻿MATLAB software. The 3D configuration was then aligned with the help of a new code using PA. As described earlier, this technique determined the centroid for each 3D configuration and scaled them to the same centroid size. Next, a reflected configuration was created by mirroring the original 3D configuration, this time about an arbitrary plane instead of the clinically derived plane. Finally, to achieve the best fit between the original 3D configuration and its reflected configuration, the latter configuration was rotated and translated over the static original configuration until the Procrustes distance (﻿sum of the squared distances between all the landmarks) was minimized (Fig. 3 c1, and d1). Finally, a region-based asymmetry score (Table 3) was computed by taking the sum of the squared Euclidean distances between each pair of landmarks and dividing by the total number of landmarks in that region [2, 29]. ﻿

### Modified Procrustes analysis (MPA)

Additional MATLAB code was written to undertake the modified Procrustes analysis (MPA). This involved importing the 3D landmark configuration (original configuration) into MATLAB software, computing the centroid and re-scaling the configuration to a common centroid size. A reflected configuration was then created by mirroring the original version around an arbitrary plane. Next, the original configuration was kept static while rotating the reflected configuration until the ﻿sum of the squared distances between four stable landmarks (bilateral porion and orbitale) was minimized to achieve the best fit between them unlike PA, wherein all the landmarks were utilized to obtain the best fit between the original and reflected configurations (Fig. 3c2 and d2). Finally, ﻿the asymmetry score for all 9 regions was computed (Table 3). MPA was designed so that the superimposition of the original and reflected 3D configurations was based solely on 4 stable landmarks, in contrast to the Procrustes analysis (PA) method which utilized all the facial landmarks during the alignment process.

### Study error

All measurements were carried out by one investigator. Intraexaminer reliability was assessed by repeating the reorientation and landmarking procedures on 13 randomly selected CBCT images from each group (26 in total). A 2-week interval was maintained amid the first and the second alignments and landmarking procedures to minimize memory bias. ﻿ The Dahlberg formula [30] was used to calculate random error for R, A, and S coordinates separately [31].

### Statistical analysis

All statistical analyses were performed using IBM SPSS Statistics for Mac, version 25.0 (IBM Corp., Armonk, N.Y., USA). Facial asymmetry was evaluated by computing the asymmetry scores of 9 regions for all the patients. Patients’ pre-operative and post-operative measured variables were compared using a Students paired *t*-test. Similarly, an independent *t-*test was used to assess the pre- and post-operative means against controls. ﻿Probabilities of *p* < 0.05 were considered significant.

## Results

The intra-examiner reliability measurements were excellent, with a mean intraclass correlation coefficient (ICC) of 0.95 (range 0.90 to 0.99), and method error ranging from 0.03 to 0.38 mm.

### Asymmetry index (AI)-based analysis

The results of the AI comparison between different groups are summarized in Table 4. The regional evaluation of AI showed that before surgery, asymmetry was more severe at all the facial regions (zone AI, part AI, and area AI) compared to controls, specifically at the chin area (9.14 mm, *p* = 0.023), followed by the lower facial region, including the mandibular midline (7.00 mm,* p* < 0.001) and mandibular bilateral region (7.36 mm,* p* = 0.003). After surgery, substantial correction of asymmetry was noticed regarding the total facial skeleton (*p* = 0.046), total mandible (*p* = 0.018), and mandibular midline (*p* < 0.001). However, even after surgery, the asymmetry was more pronounced in the total maxilla, bilateral maxilla, and bilateral mandibular regions. Despite significant improvement in asymmetry post-operatively, the symmetry achieved was not comparable to controls at the total facial skeleton (*p* = 0.002), total mandible (*p* = 0.006), and mandibular midline (*p* < 0.001).

**Table 4:** A comparison of Asymmetry Index between different groups

	Asymmetry group				Control group				
	T0		T1		C		T0-C	T0-T1	T1-C
	Mean	SD	Mean	SD	Mean	SD	*P*	*P*	*P*
Zone AI									
Total facial skeleton	5.17	3.59	3.31	2.04	1.61	1.21	< 0.001*	0.046*	0.002*
Total maxilla	2.37	0.87	1.95	1.01	0.78	0.36	< 0.001*	0.364	0.005*
Total mandible	7.27	3.42	4.33	2.03	2.24	1.25	< 0.001*	0.018*	0.006*
Part AI									
Maxilla midline	1.53	0.78	1.11	0.12	0.91	0.06	0.013*	0.053	0.173
Maxilla bilateral	2.61	0.84	2.19	1.03	0.74	0.40	< 0.001*	0.425	0.004*
Mandibular midline	7.00	0.49	2.92	0.36	1.27	0.05	< 0.001*	< 0.001*	< 0.001*
Mandibular bilateral	7.36	3.99	4.80	2.16	2.56	1.30	0.003*	0.111	0.017*
Area AI									
Chin	9.14	3.21	4.26	2.02	1.98	1.23	0.023*	0.090	0.171
Ramus	5.75	2.78	4.11	1.88	2.32	1.39	0.013*	0.218	0.066

*T0*, presurgery; *TI*, postsurgery; *C*, control

Data are presented as the means (mm) and SD (mm)

^*^P < 0.05

### Asymmetry score based on clinically derived midline (CM)

**Fig. 4:**
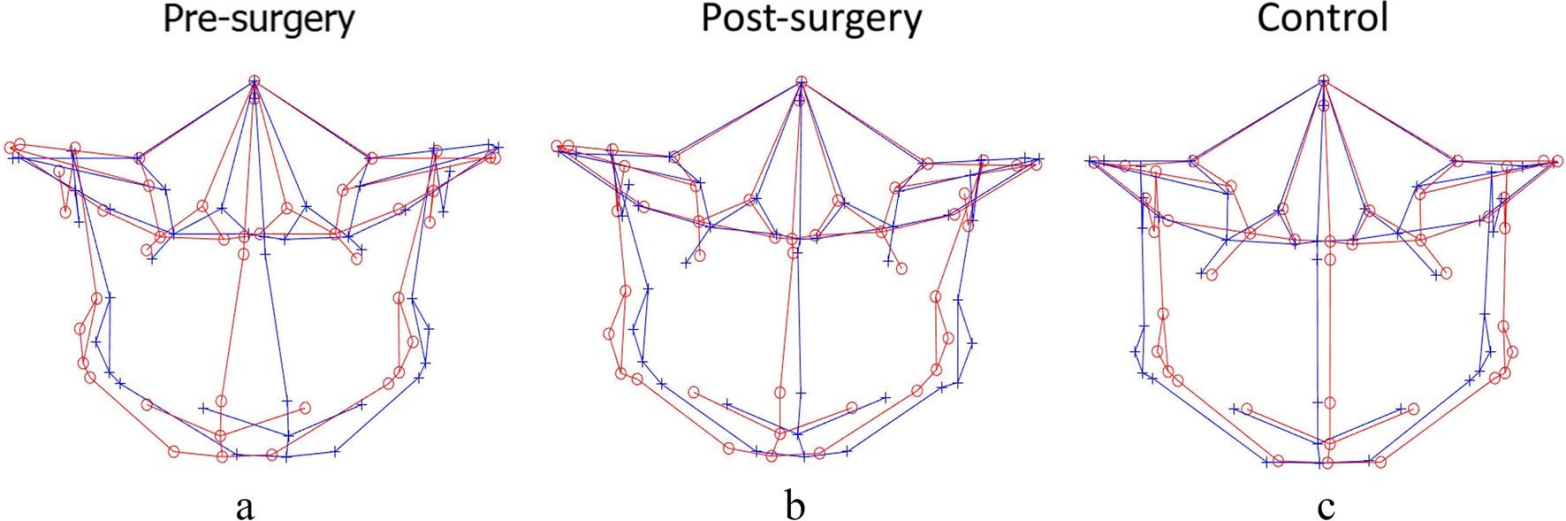
3D landmark configuration for the total facial skeleton of pre-surgery, post-surgery, and control, plotted using the clinically derived midline (CM) method (original configuration in red, reflected configuration in blue). **a**, **c** Pre-surgery asymmetry at the mandible midline, mandible bilateral, chin, and ramus region compared to controls; **a**, **b** substantial correction of mandibular asymmetry post-operatively, specifically at the mandible midline and chin region, compared to pre-surgery; **b**, **c** Post-surgery results not comparable to controls

Table 5 shows the results for the comparison of asymmetry scores using the CM method. Before surgery, asymmetry was more severe at the total facial skeleton and total mandible (*p* < 0.001 and < 0.001, respectively; zone AS), mandible midline and mandible bilateral (*p* < 0.001 and *p* < 0.001, respectively; part AS), chin and ramus (*p* < 0.001 and *p* < 0.001, respectively; area AS), as assessed by higher mean asymmetry scores compared to C (Fig. 4a). Significant correction of mandibular asymmetry was observed in the total facial skeleton (*p* = 0.020) and total mandible (*p* = 0.010) post-surgery when asymmetry scores were compared against *T*_0_ (Fig. 4b). Specifically, the mean asymmetry scores significantly decreased from 7.23 to 2.78 for the mandible midline (*p* < 0.001) and from 6.99 to 3.14 for the chin region (*p* < 0.001) after surgical correction. When post-surgery asymmetry scores were compared with controls, mean asymmetry scores at all regions of zone AS (total facial skeleton, *p* < 0.001; total maxilla, *p* = 0.03; and total mandible, *p* < 0.001); maxilla bilateral and mandible bilateral (*p* = 0.03 and *p* < 0.001, respectively; part AS); and ramus (*p* < 0.001; area AS) were found to be significantly higher for the post-surgery group (Fig. 4c). In addition, the *T*_1_-*C* results also revealed significant residual asymmetry at the mandible midline (2.78 ± 2.34, *p* = 0.010) and chin (3.14 ± 2.04, *p* = 0.020) regions.

**Table 5:** A comparison of asymmetry scores between different groups using clinically derived midline

	Asymmetry group				Control group				
	T0		T1		C		T0-C	T0-T1	T1-C
	Mean	SD	Mean	SD	Mean	SD	*P*	*P*	*P*
Zone AS									
Total facial skeleton	3.64	1.49	2.92	0.86	2.01	0.50	< 0.001*	0.020*	< 0.001*
Total maxilla	1.86	0.67	1.91	0.43	1.59	0.49	0.150	0.630	0.030*
Total mandible	4.93	2.40	3.66	1.37	2.40	0.79	< 0.001*	0.010*	< 0.001*
Part AS									
Maxilla midline	1.43	1.36	1.18	0.79	1.09	0.86	0.340	0.290	0.740
Maxilla bilateral	1.92	0.64	2.02	0.46	1.69	0.51	0.210	0.370	0.030*
Mandibular midline	7.23	4.75	2.78	2.34	1.32	0.85	< 0.001*	< 0.001*	0.010*
Mandibular bilateral	4.62	2.18	3.83	1.30	2.57	0.85	< 0.001*	0.070	< 0.001*
Area AS									
Chin	6.99	4.44	3.14	2.03	1.89	1.08	< 0.001*	< 0.001*	0.020*
Ramus	4.01	1.64	3.81	1.21	2.63	0.90	< 0.001*	0.550	< 0.001*

*T0*, presurgery; *TI*, postsurgery; *C*, control

Data are presented as the means (mm) and SD (mm)

^*^P < 0.05

### Asymmetry score based on Procrustes analysis (PA)

**Fig. 5:**
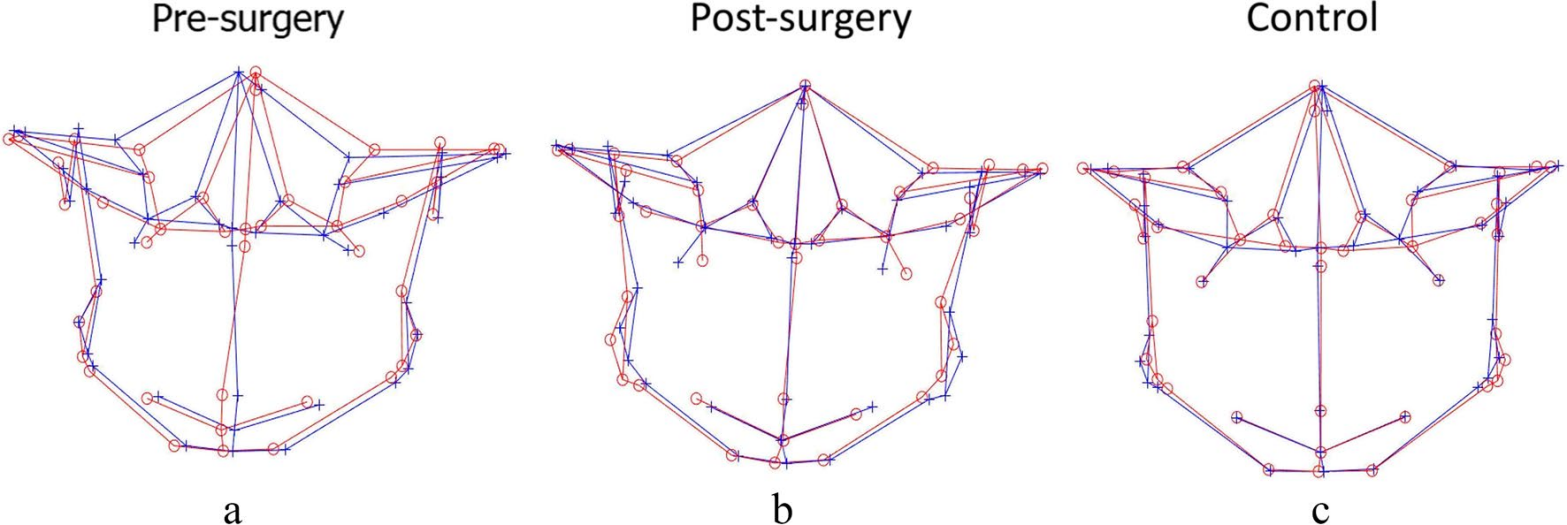
3D landmark configuration for the total facial skeleton of pre-surgery, post-surgery, and control, plotted using Procrustes analysis (PA) (original configuration in red, reflected configuration in blue). **a** Masking of asymmetry at the mandibular midline and chin region before surgery; **b** no change despite surgical correction; **c** PA masks asymmetry even in controls and represents perfect symmetry

The results for the intergroup comparison of Procrustes-derived asymmetry scores are summarized in Table 6. When asymmetry scores for different regions were compared between the pre-surgery group and controls, Procrustes failed to detect asymmetry at the mandibular midline and chin regions, while significantly higher mean asymmetry scores were noticed at the total facial skeleton and total mandible (*p* < 0.001 and *p* < 0.001, respectively; zone AS), mandible bilateral (*p* < 0.001; part AS), and ramus (*p* < 0.001; area AS) (Fig. 5a). After correction, there was no significant decrease in the asymmetry characteristics in comparison to *T*_0_; instead, the mean asymmetry scores noticed were higher in comparison to *T*_0_ for most of the regions except for the total facial skeleton and chin regions, which showed decreased asymmetry scores; however, the change was insignificant (Fig. 5b). In addition, all the regions of zone AS, including the total facial skeleton (*p* < 0.001), total maxilla (*p* = 0.02), and total mandible (*p* < 0.001); bilateral maxilla (*p* = 0.04) and bilateral mandible (*p* < 0.001) of part AS; ramus (*p* < 0.001); and area AS exhibited significantly higher mean asymmetry scores after surgery when compared with controls (Fig. 5c).

**Table 6:** A comparison of asymmetry scores between different groups using Procrustes analysis

	Asymmetry group				Control group				
	T0		T1		C		T0-C	T0-T1	T1-C
	Mean	SD	Mean	SD	Mean	SD	*P*	*P*	*P*
Zone AS									
Total facial skeleton	2.20	0.90	1.90	0.32	1.32	0.28	< 0.001*	0.12	< 0.001*
Total maxilla	1.08	0.34	1.30	0.30	1.09	0.26	0.89	0.01*	0.02*
Total mandible	1.90	0.78	1.98	0.40	1.27	0.28	< 0.001*	0.64	< 0.001*
Part AS									
Maxilla midline	0.00	0.00	0.00	0.00	0.00	0.00	0.03*	0.50	0.10
Maxilla bilateral	1.15	0.34	1.38	0.33	1.17	0.29	0.86	0.01*	0.04*
Mandibular midline	0.00	0.00	0.00	0.00	0.00	0.00	0.49	0.40	0.16
Mandibular bilateral	2.02	0.87	2.12	0.45	1.32	0.27	< 0.001*	0.64	< 0.001*
Area AS									
Chin	0.73	0.46	0.70	0.46	0.55	0.42	0.21	0.82	0.29
Ramus	1.96	0.86	2.23	0.48	1.31	0.30	< 0.001*	0.21	< 0.001*

*T0*, presurgery; *TI*, postsurgery; *C*, control

Data are presented as the means (mm) and SD (mm)

^*^P < 0.05

### Asymmetry score based on modified Procrustes analysis (MPA)

**Fig. 6:**
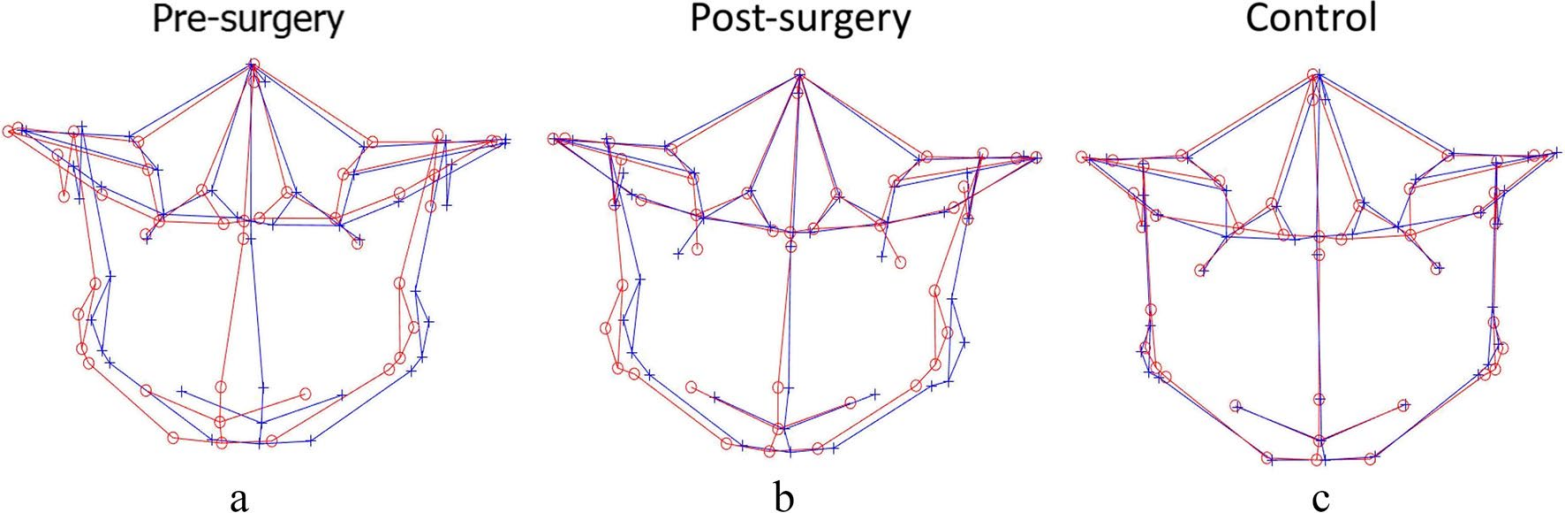
3D landmark configuration for the total facial skeleton of pre-surgery, post-surgery, and control, plotted using modified Procrustes analysis (MPA) (original configuration in red, reflected configuration in blue). **a**, **c** Pre-surgery asymmetry at the mandible midline, mandible bilateral, chin, and ramus region compared to controls; **a**, **b** substantial correction of mandibular asymmetry post-operatively, specifically at the mandible midline and chin region, compared to pre-surgery; **b**, **c** post-surgery results not comparable to controls

Pre-surgical asymmetry scores were observed to be significantly higher when matched against controls, implying severe asymmetry at all the facial regions including total facial skeleton and total mandible (*p* < 0.001 and < 0.001, respectively; zone AS), mandible midline and mandible bilateral (*p* < 0.001 and *p* < 0.001, respectively; part AS), chin and ramus (*p* < 0.001 and < 0.001, respectively; area AS) except for the maxillary region (zone AI, part AI and area AI) (Fig. 6a). Substantial improvements were noticed following surgery with respect to the lower jaw at the mandible midline (*p* < 0.001, part AS) and chin region (*p* < 0.001; area AS) compared to *T*_0_ (Fig. 6b). When post-surgical asymmetry scores were compared with controls, higher asymmetry scores were noticed at all regions except for the maxilla midline (Fig. 6c). In addition, evaluation of the surgical outcomes was indicative of persisting asymmetry at the total mandible (*p* < 0.01, zone AS), albeit significant correction. The results for the intergroup comparison of MPA-derived asymmetry scores are summarized in Table 7.

**Table 7:** A comparison of asymmetry scores between different groups using modified Procrustes analysis

	Asymmetry group				Control group				
	T0		T1		C		T0-C	T0-T1	T1-C
	Mean	SD	Mean	SD	Mean	SD	*P*	*P*	*P*
Zone AS									
Total facial skeleton	3.28	1.54	2.73	0.79	1.83	0.53	< 0.001*	0.080	< 0.001*
Total maxilla	1.76	0.62	1.85	0.62	1.53	0.47	0.180	0.610	0.070
Total mandible	4.37	2.50	3.38	1.17	2.12	0.76	< 0.001*	0.050	< 0.001*
Part AS									
Maxilla midline	1.37	1.07	1.11	1.11	1.24	0.81	0.640	0.440	0.690
Maxilla bilateral	1.81	0.59	1.96	0.63	1.59	0.47	0.200	0.340	0.040*
Mandibular midline	6.44	4.91	2.37	2.40	1.55	1.01	< 0.001*	< 0.001*	0.160
Mandibular bilateral	4.09	2.24	3.56	1.09	2.20	0.80	< 0.001*	0.250	< 0.001*
Area AS									
Chin	6.19	4.63	2.62	1.98	2.05	1.31	< 0.001*	< 0.001*	0.280
Ramus	3.53	1.68	3.61	1.02	2.16	0.76	< 0.001*	0.810	< 0.001*

*T0*, presurgery; *TI*, postsurgery; *C*, control

Data are presented as the means (mm) and SD (mm)

^*^P < 0.05

## Discussion

Matching symmetry or object symmetry defines bilateral symmetry as portrayed in biology. Matching symmetry is characterized as a structure created by 2 distinct replicas (mirror images), each located on either side of the body. ﻿Object symmetry refers to a structure that is symmetric within itself, thus devising its own internal plane of symmetry, attributable to the left and right halves as mirror images of each other [23, 32]. Two-pronged symmetry of the facial skeleton is often used during reconstructive procedures in cases with facial deformities where the unaffected side ﻿is usually used as a template for the restoration of the other. Effectual management of such cases relies on proper diagnosis and prediction for aesthetic outcomes, which again relies on the correct internal symmetry plane or midsagittal plane to which the measurements are made [8, 15, 23, 33–36]. Furthermore, the additional knowledge pertaining to the site, severity, and degree of facial deformity contributes to a favorable outcome [28]. However, a good post-surgical outcomes demand a reliable method to quantify asymmetry correctly; therefore, the results of the current study go some way toward achieving this goal.

Assessment of facial asymmetry consists of 2 steps: (1) establishing a symmetry plane that fits the asymmetric craniofacial structures and (2) gauging the degree of deviation from symmetry [37]. In an attempt to achieve an accurate diagnosis of asymmetric craniofacial structures, the present study focused on objectively quantifying the degree of asymmetry obtained using 4 different methodologies with cephalometric and morphometric modus. By comparing the outcomes of 4 different methodologies, it was found that the clinically derived midline (CM) and modified Procrustes analysis (MPA) were capable of detecting asymmetry comprehensively, especially in the mandibular region, which was analogous to AI, whereas Procrustes analysis (PA) showed contrasting results where the mandible midline and chin were symmetric. This could not be clinically valid, as skeletal distortion of the mandible and/or maxilla ﻿that sequels to Menton deviation is a chief point of concern to patients with facial asymmetry [27].

**Fig. 7:**
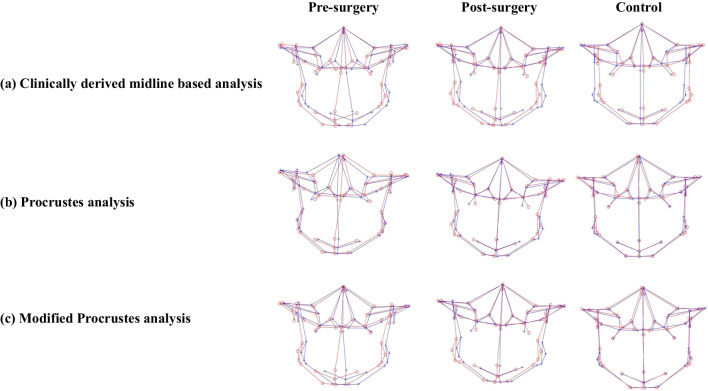
3D landmark configuration for the total facial skeleton of pre-surgery, post-surgery, and control, plotted using various quantification methods (original configuration in red, reflected configuration in blue). **a** Clinically derived midline-based analysis shows a marked correction of facial asymmetry post-surgery; **b** Procrustes analysis shows masking of asymmetry before surgery; **c** modified Procrustes analysis reflects the clinical situation more accurately and without the “masking effect”

In an effort to maintain accuracy, the same coordinate system for pre- and post-surgery craniofacial structures was used for assessing the changes following surgery to avoid the deviation of the clinical midline that could have affected the results. AI, CM, and MPA proved the surgery to be successful in decreasing asymmetric characteristics of the mandibular region, but concomitantly where CM and MPA showed substantial improvement (Fig. 7a and c), AI was unable to signify the change specific to the chin region. In contrast, PA not only failed to demonstrate changes following surgery but also calculated post-surgical asymmetry scores higher than the scores before treatment, suggesting surgery intensifies asymmetry (Fig. 7b). While other methods of quantification also revealed the fact that substantial surgical corrections for some regions could not abet in achieving norms of clinically perceived symmetric faces (controls), for example, MPA identified significant residual asymmetry at the total mandible, whereas AI identified residual asymmetry at the total facial skeleton and mandible midline in addition to the total mandible. Likewise, in addition to the aforementioned regions, CM also identified significant residual asymmetry in the chin region.

Scrutinizing the collected results of asymmetry values with different techniques showed that the results of AI, CM based on cephalometric measurements, and the new “*modified Procrustes analysis”* stemmed on morphometric analysis quadrate with each other, while PA, a morphometric method, showed quite paradoxical results (Fig. 7a–c). The underlying aim of the PA method is to minimize the sum of squared distances between the corresponding landmark configurations, i.e., to achieve the “best fit” [2]. By definition this means all the landmarks are taken into account and will therefore reduce the asymmetric characteristics [19]. This pronounced “masking effect” led to lower pre-surgery asymmetry scores, thus, lowering its clinical implications (Fig. 7b). The new modified Procrustes analysis (MPA) method only incorporates the landmarks in the upper part of the face (bilateral porions and orbitales) for “best fit,” as these are least affected by asymmetry and could be considered stable [38] (Fig. 7c).

Likewise, it can be observed that the CM shows more asymmetry than the PA and MPA methods, even for the control group, alluding to a drawback of using the CM for calculating the asymmetry score (Fig. 7a). Considering that the CM method is highly reliant on the accuracy of the midsagittal plane, which in turn is dependent on the precision with which the nasion and sella are identified, the slightest landmarking error may induce a yaw deviation in the symmetry plane (midsagittal plane) that might go undetected from the frontal view. A benefit of MPA is that it may be less sensitive to such deviations. The modified Procrustes algorithm is less “rigid” than the calculation of the clinical midline in the sense that it is the solution to a mathematical optimization problem rather than an algebraic problem.

Previous studies [4, 13, 14, 22] have confirmed the clinical legitimacy of calculating AI, and as evident from the present analysis, the quantification of asymmetry through MPA has provided outcomes that are equivalent to the results by AI. Hence, MPA is proficient in evaluating cranio-facial asymmetry with more valid clinical representation when porion and orbitale are unaffected. In the future, additional stable landmarks could be used to improve the validly and generalizability of the MPA method.

Despite meticulous evaluation of cranio-facial deformity, the retrospective nature of this study cannot be overlooked even though this limitation was minimized ﻿by selecting consecutive patients. Therefore, further studies are required to analyze craniofacial asymmetry prospectively. Additionally, this study was limited to asymmetry in class III subjects; hence, future studies analyzing asymmetry, e.g., in class II subjects or with syndromic patients, should be conducted to increase the applicability of this method.

## Conclusion

True clinical representation of asymmetry in all three dimensions is essential to effective and desirable surgical outcomes. The present study demonstrated that modified Procrustes analysis (MPA) is a promising and reliable method for the assessment of 3D facial asymmetry and is clinically applicable for class III patients seeking orthognathic surgery. Furthermore, this method has potential applications in assessing asymmetry in a wider spectrum of patients, including syndromic patients, given that symmetrical arbitrary landmarks can be located.

## Data Availability

The datasets used and/or analyzed during the current study are available from the corresponding author on reasonable request.
